# Enhanced expression of the stemness-related factors OCT4, SOX15 and TWIST1 in ectopic endometrium of endometriosis patients

**DOI:** 10.1186/s12958-016-0215-4

**Published:** 2016-11-24

**Authors:** Katharina Proestling, Peter Birner, Sukirthini Balendran, Nadine Nirtl, Erika Marton, Gülen Yerlikaya, Lorenz Kuessel, Theresa Reischer, Rene Wenzl, Berthold Streubel, Heinrich Husslein

**Affiliations:** 1 Department of Obstetrics and Gynecology, Medical University of Vienna, Waehringer Guertel 18-20, 1090 Vienna, Austria; 2 Department of Pathology, Medical University of Vienna, Waehringer Guertel 18-20, 1090 Vienna, Austria; 3 Fetal Medicine Research Institute, King’s College Hospital, 16-20 Windsor Walk, Denmark Hill, SE58BB London, UK

**Keywords:** Endometriosis, Stem cells, OCT4, SOX15, TWIST1

## Abstract

**Background:**

Current evidence suggests that endometrial-derived stem cells, spilled in the peritoneal cavity via retrograde menstruation, are key players in the establishment of endometriotic lesions. The aim of this study was to determine the presence and distribution of the stemness-related factors OCT4, SOX15, TWIST1 and DCAMLK1 in women with and without endometriosis.

**Methods:**

Immunohistochemical analysis was used to determine stromal and epithelial expression of OCT4, SOX15, TWIST1 and DCAMLK1 in endometriosis patient (EP) endometrium (*n* = 69) and endometriotic tissue (*n* = 90) and in control endometrium (*n* = 50). Quantitative Real-Time PCR of OCT4, SOX15 TWIST1 and DCAMLK1 was performed in paired samples of EP endometrium and endometriotic tissue. Co-immunofluorescence staining was performed for OCT4 and SOX15. For statistical analyses we used unpaired *t*-test, Fisher combination test and Spearman test. For paired analyses, paired *t*-test and McNemar test were used.

**Results:**

We detected a significant correlation between the expression of the established stem cell marker OCT4 and the stemness-related markers SOX15 (*p* < 0.001) and TWIST1 (*p* = 0.002) but not DCAMLK1. We showed a colocalization of SOX15 and OCT4 in epithelial and stromal cells of endometriotic tissue by coimmunofluorescence. A concordant expression of OCT4 and SOX15 in the same sample was observed in epithelial cells of the endometriotic tissue (71.7%). The expression of stemness-related factors was not associated with proliferative or secretory phase of the menstrual cycle in endometriosis patients but was found to be differentially expressed during the menstrual cycle in the control group. Increased expression of epithelial OCT4, SOX15 and TWIST1 was detected in endometriotic tissue compared to EP endometrium in paired (*p* = 0.021, *p* < 0.001 and *p* < 0.001) and unpaired analysis (*p* = 0.040, *p* < 0.001 and *p* = 0.001).

**Conclusion:**

Our findings support the hypothesis that upregulation of stem cell-related factors contribute to the establishment of endometriotic lesions.

**Trial registration:**

The study was approved by the institutional review board (545/2010 on 6th of May 2014) of the Medical University of Vienna (http://ethikkommission.meduniwien.ac.at/fileadmin/ethik/media/dokumente/register/alle_2010.pdf).

**Electronic supplementary material:**

The online version of this article (doi:10.1186/s12958-016-0215-4) contains supplementary material, which is available to authorized users.

## Background

Endometriosis is a benign gynecological disease, which is characterized by the presence of functional endometrial glands and stroma outside the uterine cavity [1]. The exact aetiology of endometriosis is unclear. One widely accepted hypothesis is that endometriosis originates from retrograde menstruation of endometrial cells which implant on peritoneal surfaces [2]. During the reproductive life of women the endometrium undergoes profound changes according to the different phases of the menstrual cycle [3]. Several studies revealed the presence of adult stem cells in the basalis layer as well as functionalis layer of the human endometrium [4–7]. These endometrium-derived stem cells, which are distributed by retrograde menstrual efflux, may contribute to the establishment of ectopic endometriotic lesions [7–11]. The monoclonal origin of some endometriotic lesions, long-time culture properties of cell clones established from endometriotic lesions and the isolation of progenitor cells from menstrual blood support this hypothesis [12–17].

Recent studies have been evaluating candidate markers for endometrial progenitor stem cells. Several general adult stem cell markers, such as bcl-2, c-kit (CD117), CD34 and noteworthy OCT4 have been identified in human endometrial tissue samples [4, 7, 18–22]. The transcription factor OCT4 is crucial for the maintenance of cell pluripotency and is known to be expressed in embryonic stem cells, germ cells and in adult stem cells [7, 23]. Furthermore, in vitro studies have shown that elevated expression of OCT4 and SOX2 in human endometrial cells contributed to reprogramming these cells into induced pluripotent stem cells (iPS cells) suggesting that OCT4 and SOX2 are stemness-related factors in human endometrium [24]. In endometriosis, the epithelial expression of OCT4 evaluated by immunohistochemistry was significantly higher in ectopic lesions compared to eutopic endometrium of patients with and without endometriosis. [25, 26]. Although the transcription factor OCT4 is a known key modulator for stem cell properties of primate stem cells, it further forms complexes with many different partners and displays various functions [27]. Several SOX-OCT transcription factor combinations have been identified and the cooperation correlates with the efficiency in producing iPS cells [28]. SOX15 for example strongly cooperates with OCT4 on the canonical pathway and has not been investigated in endometriosis so far [28].

The aim of this study was to analyze the role of stem cell-related markers in endometriosis. We correlated the expression of SOX15 and the potential stem cell factors TWIST1 (twist family bHLH transcription factor 1) and DCAMLK1 (doublecortin- and calmodulin kinase-like 1) with OCT4 expression. We analyzed the coexpression of SOX15 and OCT4 in epithelial and stromal cells of endometriotic lesions. Further we investigated the expression of stemness-related factors during the proliferative and secretory phase of the menstrual cycle, in patients with and without endometriosis and in different stages of endometriosis.

## Methods

### Patients and tissue samples

Tissue samples were obtained from 160 premenopausal women (mean age 34.5 ± 6.4 years), who underwent surgery at the General Hospital of Vienna between 2010 and 2014 due to the suspicion of endometriosis with or without infertility, chronic pelvic pain, benign adnexal masses, or uterine leiomyoma. The tissue samples have been collected in the context of a prospective cohort study called EMMA Study (EndoMetriosisMarkerAustria). The 160 cases consisted of 110 patients with endometriosis and 50 control patients who underwent laparoscopy and endometrial sampling with or without hysteroscopy due to benign adnexal masses, chronic pelvic pain or uterine fibroids. Exclusion criteria were pregnancy or breastfeeding less than 6 months prior to the beginning of the study, malignant disease, infectious diseases (HIV, hepatitis A, B and C, tuberculosis) and autoimmune diseases. Among the 110 cases with endometriosis, we obtained matched samples of endometriotic and eutopic endometrium in 49 cases, exclusively endometriosis patient (EP) endometrium in 20 cases, and exclusively endometriotic tissue in 41 cases. The study was approved by the institutional review board (545/2010) of the Medical University of Vienna. All participants provided written informed consent. The matched sample tissues were collected during the same surgical procedure. Tissues were sliced in two. One part of the sample was snap-frozen in liquid nitrogen (LN2) immediately after surgical extraction and stored at -80° Celsius to minimize enzymatic degradation until final analysis. The other part of the tissue was fixed in 4% buffered formalin immediately after surgical extraction and stored overnight for processing of immunohistochemical analysis. Endometriosis was diagnosed histologically in all endometriosis patients. Staging was performed according to the revised American Fertility Society (rAFS) classification guidelines (I, *n* = 17; II, *n* = 23; III, *n* = 22; IV, *n* = 25) [29]. Cycle phase was determined by histopathological examination of endometrial samples by an experienced pathologist and was confirmed by date of last menstrual period.

Characteristics of the study populations are provided in Table 1.

**Table 1:** Description of the study population

					Endometriosis patients (EP, n = 110)			
		Total	Control endometrium (*n* = 50)		Eutopic endometrium (*n* = 69)		Endometriotic tissue (*n* = 90)	
Age (years)		209	34.9 ± 5.8		33.1 ± 6.1		34.7 ± 6.8	
Cycle Phase	Proliferative	99	24	(48.0%)	31	(44.9%)	44	(48.9%)
	Secretory	92	20	(40.0%)	37	(53.6%)	35	(38.9%)
	na	18	6	(12.0%)	1	(1.4%)	11	(12.2%)
Staging	I or II	62			22	(31.9%)	40	(44.4%)
	III or IV	72			25	(36.2%)	47	(52.2%)
	na	25			22	(31.9%)	3	(3.3%)

*EP* Endometriosis patient. Numbers of patients in each of the indicated subgroups are shown. Numbers in parentheses indicate the fraction of patients (%) in each column in proliferative and secretory cycle phase or with low and high stage [28]. na, status not available

### Quantitative Real-Time PCR (qRT-PCR)

Briefly, total RNA was isolated from fresh frozen tissues with the Absolutely RNA miRNA Kit (Agilent, CA, USA) and reverse-transcribed with the SuperScript First-Strand Kit (Invitrogen, CA, USA) according to the manufacturers’ instructions. Each sample was analyzed by real-time PCR on an Applied Biosystems 7500 fast instrument, using gene-specific primers and fluorescent probes obtained from Applied Biosystems (CA, USA): OCT4, Hs00999632_g1; SOX15, Hs_00199511_m1; TWIST1, Hs_01675818_m1; DCAMLK1, Hs00178027_m1; GAPDH (control), Hs_99999905_m1, and ACTB (control), Hs_99999903_m1. The mRNA levels of OCT4, SOX15, TWIST1 and DCAMLK1 were normalized to those of ACTB and GAPDH in each sample by subtracting the mean Ct (threshold cycle) values of the controls from the Ct value of OCT4, SOX15,TWIST1 and DCAMLK1 as described previously [30]. For binary analysis, the cutoff was set at the median levels for SOX15 and TWIST1 expression.

### Immunohistochemistry (IHC)

Immunohistochemical staining was performed on formalin-fixed, paraffin-embedded tissues. Three-micrometer thick sections were cut and placed on glass slides. Heat antigen retrieval was performed in 10mM Sodium Citrate Buffer pH6. Nonspecific background staining was blocked by incubating in H_2_O_2_ and with Ultra V Block (Thermo Scientific, Ultra Vision LP Kit, TL-060-HL, MA, USA) according to the protocol. The following antibodies were used: the rabbit polyclonal anti-TWIST antibody (Abcam, ab50581, Cambridge, UK) was applied at a dilution of 1:1200 with Antibody Diluent with Background Reducing Components (Dako, S3022, Glostrup, Denmark), the mouse monoclonal anti-DCAMKL1 antibody (Abcam, ab88484, Cambridge, UK) at a dilution of 1:500, the rabbit monoclonal anti-OCT4 antibody (Abcam, ab109183, Cambridge, UK) at a dilution of 1:500, and the rabbit polyclonal anti-SOX15 (Abcam, ab55960, Cambridge, UK) at a dilution of 1:300. Ultra Vision LP Kit was used for detection according to the protocol (Thermo Scientific, Ultra Vision LP Kit, TL-060-HL, MA, USA). Finally, all slides were incubated with DAB-Substrate (Dako, K346811, Glostrup, Denmark) and counterstained in Hematoxylin before they were dehydrated and mounted.

### Scoring and Immunohistochemical Analysis

**Fig. 1:**
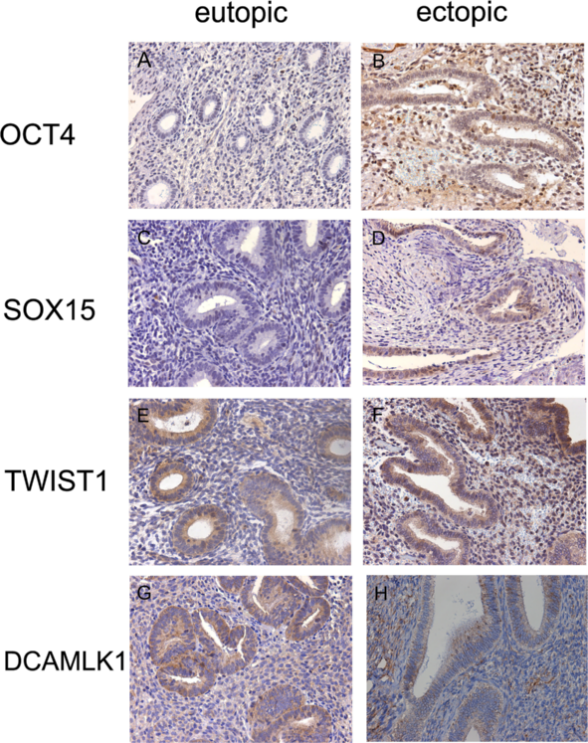
Immunohistochemical analyses of OCT4, SOX15, TWIST1 and DCAMLK1 in eutopic and ectopic endometrium. Anti-OCT4 and anti-SOX15 antibodies were applied at a dilution of 1:500 and 1:300, respectively, and yielded nuclear staining in eutopic (**a, c**) or ectopic tissue (**b, d**). Anti-TWIST1 antibody was applied at a dilution of 1:1200 and yielded cytoplasmatic and nuclear staining in eutopic (**e**) and ectopic (**f**) lesions. For evaluation, only nuclear staining was analyzed. Anti-DCAMLK1 antibody was applied at a dilution of 1:500 and yielded cytoplasmatic staining in eutopic (**g**) or ectopic tissue (**h**). Magnification = 200x

Prior to immunohistochemistry, endometriotic lesions consisting of well-defined glandular epithelial and stromal cells were identified in hematoxylin-eosin stained sections by a pathologist. Serial sections were cut from the chosen samples. A semiquantitative subjective scoring system to evaluate the localization, quantity and intensity of immunoreactivity was employed using light microscopy (200x magnification). In each sample, the staining for glandular epithelial cells and stromal cells was scored separately. The intensity of the staining was scored using a four-point scoring scale (0, negative staining; 1, weak staining; 2 moderate staining, 3, strong staining). The percentage of positively stained cells was again scored by a four-point scoring scale (0, negative staining; 1, 1-35% positive cells; 2, 36-70% positive cells; 3, >67% positive cells). The two scores were combined by multiplication to derive a final IHC score (0-9). For binary analysis, the cutoff was set at the median level of the final IHC score. Evaluations were performed in blind by two investigators. Positive (Seminoma) and negative (without primary antibody) controls were run concurrently. OCT4 (Fig. 1a, b) and SOX15 proteins (Fig. 1c, d) were expressed in the nucleus of the epithelial and the stromal cells of eutopic and ectopic endometrium. TWIST1 expression was observed in the cytoplasm and nucleus of epithelial and stromal cells (Fig. 1e, f). However, as a transcription factor, activated TWIST1 exerts its main function in the nucleus. Thus, for TWIST1, only nuclear staining of epithelial and stromal cells was evaluated. DCAMLK1 protein was expressed in the cytoplasm of the epithelial and stromal cells in eutopic and ectopic endometrium (Fig. 1g, h).

### Confocal Immunofluorescence Studies

Immunofluorescence staining was performed on formalin-fixed, paraffin-embedded tissue. Heat Antigen Retrieval was performed in 10mM Sodium Citrate Buffer pH6. Nonspecific background staining was blocked by incubating in 0.05% fish skin in PBS. The rabbit polyclonal anti-SOX15 antibody (Abcam, ab55960, Cambridge, UK) and the mouse monoclonal anti-OCT4 antibody (Abcam, ab184665, Cambridge, UK) were applied at a dilution of 1:100 and incubated over night at 4°C. Secondary antibodies from Alexa (life technologies CA, USA, goat anti-mouse IgG: Fluor 546 (red), A-11018, goat anti-rabbit IgG, Fluor 488 (green), A11070) were diluted 1:1000 with 0.05% fish skin and incubated with 1μg/ml DAPI at room temperature for one hour. The slides were mounted with Fluoromount-G (Southern Biotech 0100-01, AL, USA). Colocalization of SOX15 and OCT4 was analyzed at 630x and 945x magnification using a Zeiss LSM 700 photomicroscope and LSM software (Zeiss, Göttingen, Germany).

### Statistical analysis

Data were analyzed using SPSS (17.0, IBM Corp., NY, USA). For correlation analysis, Spearman test was used. For paired statistics, the McNemar test and paired t-test were used. Unpaired Student t-test was used to compare two groups. To account for repeated measures we compared expression differences between EP endometrium and endometriotic tissue using separate tests in the paired (paired t-test) and unpaired patient subsets (Welch t-test). To obtain a p-value for the overall comparison between EP endometrium and endometriotic tissue the resulting p-values were combined using Fisher combination test [31]. Analyses for the Fisher combination tests were performed using R software (R-project.org). We consider the subgroup analyzes as exploratory, and hence did not adjust for multiple testing, as recommended by Bender and Lange [32]. Statistical significance was defined as *p* < 0.05.

## Results

### Increased OCT4 expression in epithelium of endometriotic tissue

**Fig. 2:**
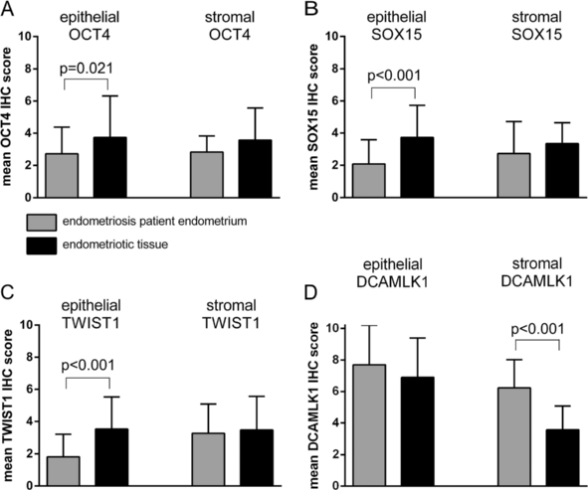
Expression analyses in 49 paired cases with endometriosis. IHC was used to analyze the protein expression of OCT4 (**a**), SOX15 (**b**), TWIST1 (**c**) and DCAMLK1 (**d**). Results are expressed as mean score of the immunohistochemical staining ± SD. Epithelial and stromal expression was analyzed separately. All p-values of paired comparisons were analysed by paired *t*-test

**Fig. 3:**
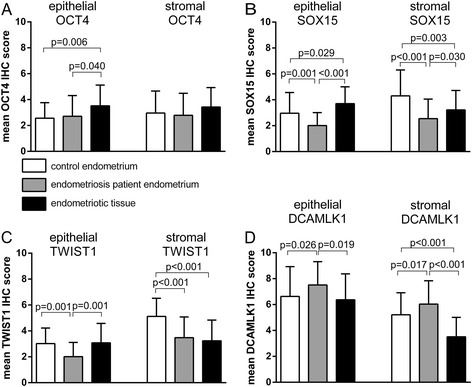
Expression analyses in 50 control patients, and 110 patients with endometriosis (eutopic and ectopic). IHC was used to analyze the protein expression of OCT4 (**a**), SOX15 (**b**), TWIST1 (**c**) and DCAMLK1 (**d**). Results are expressed as mean score of the immunohistochemical staining ± SD. Epithelial and stromal expression was analyzed separately. Comparisons with the control group were analysed by *t*-test, comparisons between EP endometrium and endometriotic tissue were performed using Fisher combination test

**Fig. 4:**
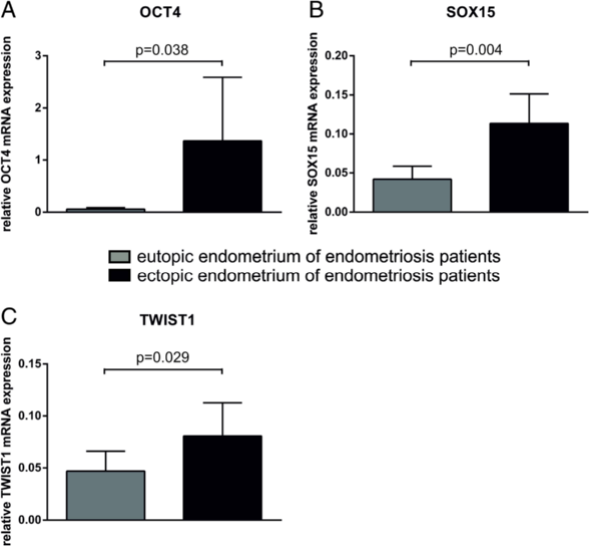
Relative mRNA expression levels in 49 paired cases with endometriosis. Quantitative Real Time PCR was used to analyze the mRNA expression levels of OCT4 (**a**), SOX15 (**b**), and TWIST1 (**c**). Expression levels were normalized to ß-actin and GAPDH. Expression levels were shown as mean ± SD. All p-values were analysed by paired *t*-test

In a first step we investigated the OCT4 expression in EP endometrium and endometriotic tissue. Analysis of the 49 endometriosis cases with paired samples (i.e. concomitant EP endometrium and endometriotic tissue) demonstrated a significant increase of OCT4 in glandular epithelium of endometriotic tissue (*p* = 0.021; paired *t*-test, Fig. 2a). No significant difference was found in the stroma (Fig. 2a). In a next step we compared the samples of the whole cohort of 110 endometriosis patients (including unpaired cases) with the 50 control cases. Similarly, epithelial OCT4 expression was significantly increased in endometriotic tissue compared to EP endometrium (*p* = 0.040, Fisher combination test, Fig. 3a) and control endometrium (*p* = 0.006, *t*-test, Fig. 3a). The stroma showed no significant differences (Fig. 3a). Similar to the epithelial protein expression of OCT4, OCT4 mRNA expression was significantly increased in endometriotic tissue compared to EP endometrium of paired samples (*p* = 0.038; paired *t*-test, Fig. 4a). Therefore we concluded that OCT4 is overexpressed in the epithelium of endometriotic tissue.

### Increased SOX15 expression in epithelium of endometriotic tissue

Next we analyzed SOX15 expression. Similar to OCT4, SOX15 expression was significantly increased in the epithelium of endometriotic tissue compared to EP endometrium (paired samples *p* < 0.001; paired *t*-test, Fig. 2b; whole cohort including unpaired samples *p* < 0.001; Fisher combination test, Fig. 3b). SOX15 was also significantly increased in the epithelium of endometriotic tissue compared to control endometrium (*p* = 0.029; *t*-test, Fig. 3b). The analysis of stromal SOX15 expression revealed no significant difference in paired samples (Fig. 2b). In contrast to the paired samples, significant differences were found for stromal SOX15 when investigating all cases (endometriotic tissue vs EP endometrium *p* = 0.030, Fisher combination test, Fig. 3b; EP endometrium vs control endometrium *p* < 0.001, *t*-test, Fig. 3b; endometriotic tissue vs. control endometrium *p* = 0.003, *t*-test, Fig. 3b). Similar to the epithelial protein expression of SOX15, SOX15 mRNA expression was significantly increased in endometriotic tissue compared to EP endometrium of paired samples (*p* = 0.004; paired *t*-test, Fig. 4b; *p* = 0.041; McNemar, Table 2). We concluded that - similar to OCT4 – SOX15 is significantly overexpressed in the epithelium of the endometriotic tissue.

**Table 2:** SOX15 mRNA expression in EP endometrium and endometriotic tissue of the same patient

		SOX15 mRNA in endometriotic tissue					
		Total	neg		pos		*P*-value
SOX15 mRNA in EP endometrium	neg	17	2	(11.8%)	15	(88.2%)	0.041
	pos	12	5	(41.7%)	7	(58.3%)	

Numbers of patients in each of the indicated subgroups are shown. Numbers in parentheses indicate the fraction of patients (%) in each row in ectopic endometriotic lesions negative and positive for SOX15. All p-values of subgroup comparisons were analysed by McNemar Test

### Increased TWIST1 expression in epithelium of endometriotic tissue

The analysis of TWIST1 revealed that TWIST1 expression was significantly increased in the epithelium of endometriotic tissue (paired samples *p* < 0.001; paired *t*-test, Fig. 2c; all endometriosis patients including the unpaired samples *p* = 0.001; Fisher combination test, Fig. 3c). The analysis of stromal TWIST1 expression revealed no significant differences between endometriotic tissue and EP endometrium in paired and unpaired analysis (Fig. 2c, Fig. 3c). Further a higher stromal TWIST1 expression was found in the control endometrium compared to EP endometrium and endometriotic tissue (both *p* < 0.001, Fig. 3c). A higher epithelial TWIST1 expression was found in the control endometrium compared to EP endometrium (*p* = 0.001, Fig. 3c). Similar to the epithelial protein expression of TWIST1, TWIST1 mRNA expression was significantly increased in endometriotic tissue compared to EP endometrium of paired samples (*p* = 0.029; paired *t*-test, Fig. 4c).

In conclusion, epithelial TWIST1, SOX15, and OCT4 are overexpressed in endometriotic tissue compared to EP endometrium of paired and unpaired endometriosis samples. No significant differences were found in the stroma of the paired endometriosis cases.

### Reduced DCAMLK1 expression in endometriotic tissue

In the analysis of endometriosis patients, epithelial and stromal DCAMLK1 expression was found to be significantly reduced in endometriotic tissue compared to EP endometrium in paired (stromal: *p* < 0.001; paired *t*-test, Fig. 2d) and unpaired samples (epithelial: *p* = 0.019, stromal: *p* < 0.001; Fisher combination test, Fig. 3d). In the comparison of endometriosis and control patients, epithelial and stromal DCAMLK1 expression was significantly increased in EP endometrium (epithelial: *p* = 0.026; stromal: *p* = 0.017, *t*-test, Fig. 3d). The mRNA levels of DCAMLK1 determined by qRT-PCR were very low in all samples and occasionally below detection level (Additional file 1). We concluded that DCAMLK1 is down-regulated in ectopic endometrium. This is in contrast to the findings for OCT4, SOX15, and TWIST.

### OCT4, SOX15 and TWIST1 but not DCAMLK1 expressions are positively correlated in endometriotic tissue

Next we correlated the four markers. A positive correlation was observed between epithelial OCT4 and epithelial SOX15 expression in endometriotic tissue (*p* < 0.001, Spearman’s rho 0.434, Table 3). Epithelial OCT4 expression also significantly correlated, although to a lower degree, with epithelial TWIST1 expression in endometriotic tissue (Spearman’s rho 0.336, *p* = 0.002, Table 3). A significant but very low correlation was also shown between ectopic epithelial SOX15 and TWIST1 expression (*p* = 0.019, Spearman’s rho 0.253, Table 3). No significant correlations were shown between DCAMLK1 and OCT4, SOX15 or TWIST1 in endometriotic tissue (Table 3). Thus we concluded that OCT4 predominately correlated with SOX15 expression.

**Table 3:** Spearman’s correlation between the epithelial expression of OCT4, SOX15, TWIST1 and DCAMLK1 in endometriotic tissue

		OCT4	SOX15	TWIST1	DCAMLK1
OCT4	Corr. Coefficient	1	**0.434**	**0.336**	0.078
	Sig. (2-tailed)		**2.63 × 10** ^**-5**^	**0.002**	0.477
	n	88	87	85	85
SOX15	Corr. Coefficient		1	**0.253**	0.072
	Sig. (2-tailed)			**0.019**	0.513
	n		89	85	86
TWIST1	Corr. Coefficient			1	0.14
	Sig. (2-tailed)				0.204
	n			86	84
DCAMLK1	Corr. Coefficient				1
	Sig. (2-tailed)				
	n				87

Expression of all factors was determined by immunohistochemistry. Correlation Coefficients and *p*-values with statistically significant *p*-values were emphasised Bold.

### Concordant expression of OCT4 and SOX15

We analyzed the concurrent expression of OCT4 and SOX15. 71.7% (33/46) of endometriotic samples of the endometriosis cohort showing positive epithelial expression of OCT4 simultaneously showed positive epithelial staining for SOX15 (data not shown). In stromal cells of endometriotic tissue, 57.8% (26/45) of the samples with positive OCT4 staining also showed positive SOX15 expression (data not shown). There was no significant difference between SOX15 and OCT4 expression in the epithelium (*p* = 0.584, McNemar) or stroma (*p* = 0.643, McNemar) analyzed in the same endometriotic tissue sample (data not shown).

**Fig. 5:**
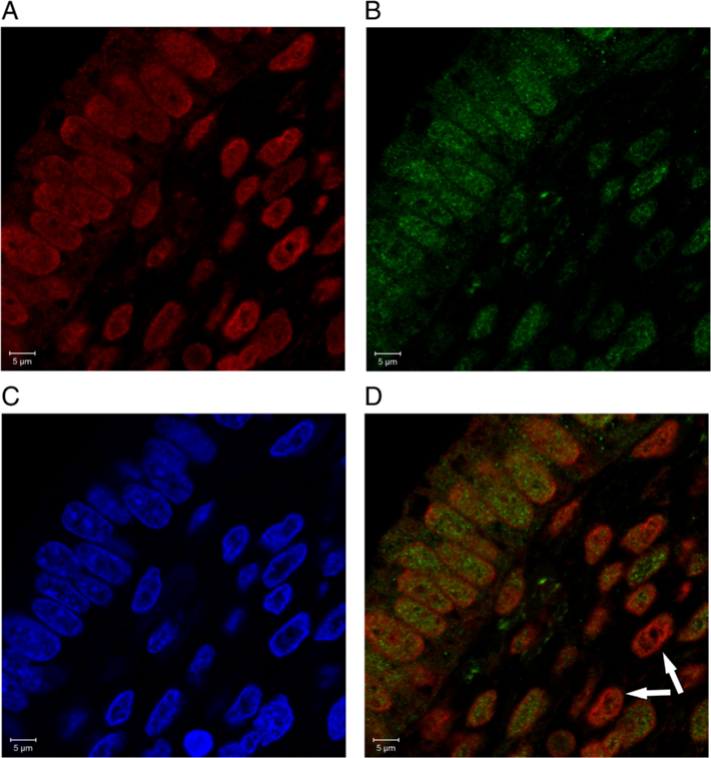
Colocalization of OCT4 and SOX15 in human endometriotic tissue. Paraffin sections of human endometrium were processed for immunofluorescence microscopy. OCT4 (**a**), SOX15 (**b**), and DAPI (**c**) stainings are shown. OCT4/SOX15 merged picture (**d**) denotes colocalization in all epithelial cells whereas few stromal cells were only positive for OCT4 (white arrows)

Confocal immunofluorescence microscopy revealed nuclear staining of epithelial SOX15, which mostly colocalized with epithelial OCT4. Some stromal cells showed OCT4 staining without SOX15 expression (white arrows, Fig. 5). Similarly to OCT4 and SOX15 expression, TWIST1 and SOX15 mRNA were also expressed concordantly. Best concordance was observed in endometriotic tissue where 73.8% (31/42) of SOX15 mRNA positive samples were also positive for TWIST1 mRNA expression (data not shown). There was no significant difference between SOX15 and TWIST1 mRNA expression in the same endometriotic tissue sample (*p* = 0.839, McNemar, data not shown).

### Expression of epithelial OCT4, SOX15 and TWIST1 does not correlate with cycle phase in patients with endometriosis nor with endometriosis staging

**Fig. 6:**
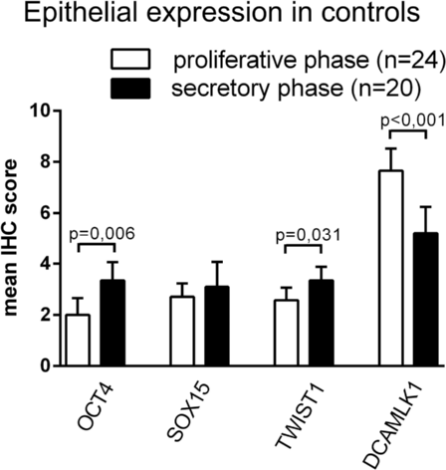
Expression levels of epithelial OCT4, SOX15, TWIST1 and DCAMLK1 in proliferative and secretory phase of the menstrual cycle in control patients. Results are expressed as mean scores of the immunohistochemical stainings ± SD. All p-values of subgroup comparisons were analysed by *t*-test

No significant difference in OCT4, SOX15, TWIST1 or DCAMLK1 expression was observed between proliferative and secretory phase neither in EP endometrium nor in endometriotic tissue (data not shown). In contrast, in control endometrium epithelial expression of DCAMLK1 is higher in patients in proliferative cycle phase than in secretory phase (*p* < 0.001, *t*-test, Fig. 6). The expression levels of epithelial TWIST1 and OCT4 are significantly reduced in the proliferative phase of control patients (*p* = 0.031 and *p* = 0.006, *t*-test, Fig.6). The epithelial expression of SOX15 is also reduced in proliferative phase of control patients, however at a non-significant level (Fig. 6).

No significant difference in OCT4, SOX15, TWIST1 or DCAMLK1 expression was observed between the different stages of endometriosis according to the rAFS classification.

## Discussion

In the present study we document the possible importance of stem cells for the development of endometriosis through the analysis of four stemness-related markers in EP endometrium, control endometrium and endometriotic tissue. Functional analyses of stem cell properties in endometriotic cells are quite limited. However, elevated expression of OCT4 and SOX2 in human endometrial cells contributed to reprogramming these cells into pluripotent stem cells (iPS cells) suggesting that OCT4 and SOX2 are stem cell factors in human endometrium [24]. SOX-OCT transcription factor combinations correlate with the efficiency in producing iPS cells. Thus we also analyzed a potential correlation and coexpression of OCT4 and SOX15, since SOX15 protein expression has never been studied in endometriosis [28]. We studied OCT4 and SOX15 expression in 110 cases and confirmed a positive correlation and overexpression of both factors in the epithelium of endometriotic tissue. Our findings are in line with previous studies with smaller sample sizes reporting an increased expression of stemness-related markers in endometriosis [25, 26]. SOX2 expression has been shown to be significantly increased in endometriosis compared to secretory endometrium of patients without endometriosis [33]. Musashi-1, an epithelial progenitor cell marker regulating self-renewal pathways, was also found to be overexpressed in endometriotic tissue compared to eutopic endometrium of controls [34]. SALL4, which is a transcriptional regulator of OCT4 and a marker for pluripotency, has been shown to be expressed in endometriotic tissue but not in EP endometrium [26]. Interestingly, SOX15 and TWIST1 expression was lower in EP endometrium compared to the control endometrium. These factors may be upregulated in endometrial cells after dissemination to promote cell survival and invasion predominantly outside the uterine cavity in order to form endometriotic lesions.

The enhanced expression of stemness-related markers in endometriotic tissue may foster self-renewal and increase cell survival. Analysis of eutopic endometrial cells collected from the menstrual blood of patients with and without endometriosis revealed that endometriosis patients had higher mRNA expression of OCT4 and SOX2 than normal controls [35]. Furthermore, women with endometriosis have larger volumes of retrograde menstrual flow than women without endometriosis and baboons whose cervices had been ligated showed a significant increase in development of endometriosis [36, 37]. Thus, our findings may support the hypothesis that cells with stem cell capacity, disseminated into the peritoneal cavity via retrograde menstrual efflux, may accumulate and contribute to the establishment of ectopic endometriotic lesions [7–11]. However, another proposal concerning the histologic origin of endometriosis is that circulating blood cells originating from bone marrow can differentiate into endometriotic tissue at various sites [38]. According to this, a small number of the accumulated stem cells shown in the endometrium of endometriosis patients may also derive from bone marrow. Moreover, it is unclear if increased stem cell number or even abnormal stem cell properties facilitate the establishment of ectopic endometrial implants in patients developing endometriosis.

We and others found an overexpression of TWIST1 in ectopic endometrium [39, 40]. TWIST1 is a marker for epithelial to mesenchymal transition (EMT), and increases the migratory activity of endometrial cells [39]. Recent evidence suggest that cells undergoing EMT acquire stem cell-like properties and the mesenchymal status seems to be a condition to regain pluripotency [41]. Thus, TWIST1 could be another potential stemness-related marker in human endometrium.

DCAMLK1, a microtubule-associated kinase, is a putative stem cell marker in pancreas and intestine, which regulates TWIST1, MYC, KRAS and other factors. DCAMLK1 was identified as a novel pancreatic cancer stem cell marker [42]. Interestingly, DCAMLK1 has the capacity to distinguish between normal and tumor stem cells at least in the intestine [43]. So far, no study has analyzed the expression of DCAMLK1 in endometriosis. Surprisingly, we found significant results for DCAMLK1 with an ectopic expression pattern inverse to OCT4. Stromal and epithelial DCAMLK1 expression was significantly higher in EP endometrium than in endometriotic tissue. The expression of stromal and epithelial DCAMLK1 was significantly increased in EP endometrium compared to control endometrium. This finding suggests that in endometriosis patients DCAMLK1 is upregulated in cells of the uterine cavity before dissemination into the peritoneal cavity. The upregulation of DCAMLK1 in EP endometrium may be due to enhanced posttranslational stabilization of the protein as the mRNA levels of DCAMLK1 were very low. Although, it cannot be excluded that the diverse expression pattern of DCAMLK1 is unrelated to stem cell properties, our findings may indicate that eutopic upregulation of DCAMLK1 may be of importance for endometriotic cell survival before the establishment of endometriotic lesions.

The correlation of stem cell factors with menstrual cycle is controversial. It has been reported that the number of clonogenic epithelial and stromal cells did not vary between proliferative and secretory endometrium in women without endometriosis [6]. Furthermore, most studies did not find a correlation between the expression of stemness-related factors and the phase of the menstrual cycle [19, 20, 26, 34]. This is in concordance with our findings showing no correlation between the epithelial or stromal expression of the stemness-related markers DCAMLK1, OCT4, SOX15 and TWIST1 and the cycle phase of endometriosis patients, neither in EP endometrium nor in endometriotic lesions. In contrast, few studies found an increased expression of stemness-related markers, such as SOX2 or Musashi-1, in proliferative phase [33, 34]. However, in patients without endometriosis, we found significantly higher epithelial expression of DCAMLK1 in the proliferative cycle phase than in the secretory phase whereas the expression levels of epithelial OCT4, SOX15 and TWIST1 are rather reduced in the proliferative phase of patients without endometriosis. Therefore, it has to be pointed out that some differences of the expression levels of stemness-related factors between endometrium of controls and patients may be hormonally related. Generally, an upregulation of stemness-related factors in normal endometrium possibly prepares the tissue for renewal by accumulation of pluripotent cells in the end of the menstrual cycle.

Lastly, it must be mentioned that even for the established stem cell marker OCT4 a formal proof of stem cell property is still lacking in endometriotic cells. Thus in endometriotic cells colony forming assays after transduction or knockdown of these stemness-related factors would be of great interest.

## Conclusion

In conclusion, the results reported herein suggest that in endometriosis SOX15 and TWIST1 may be stemness-related markers as their expression correlates with OCT4 expression. The epithelial expression of OCT4, SOX15 and TWIST1 is increased in endometriotic tissue compared to EP endometrium in paired and unpaired analysis supporting the hypothesis that upregulation of stem cell-related markers contribute to the establishment of ectopic endometriotic lesions.

## Additional file

Additional file 1: Quantitative Real Time PCR was used to analyze the mRNA expression levels of DCAMLK1. Representative qRT-PCR results (Ct values) for DCAMLK1 and control genes (ACTB and GAPDH) are shown. (DOCX 216 kb)

## Abbreviations

bcl-2: B-cell cll/lymphoma 2
CD34: Hematopoietic progenitor cell antigen CD34
c-kit: Kit, V-kit Hardy-Zuckerman 4 feline sarcoma viral oncogene homolog, cd117
DCAMLK1: Doublecortin- and calmodulin kinase-like 1
EM: endometriosis
EMT: epithelial to mesenchymal transition
iPS cells: induced pluripotent stem cells
KRAS: V-KI-RAS2 Kirsten rat sarcoma viral oncogene homolog
Musashi-1: Musashi, drosophila, homolog of, 1
MYC: V-MYC Avian myelocytomatosis viral oncogene homolog
na: status not available
OCT4: Octamer-binding transcription factor 4
qRT-PCR: quantitative Real-Time Polymerase Chain Reaction
rAFS: revised American fertility society
SALL4: Sal-Like 4
SOX15: Sry-box 15
SOX2: Sry-Box 2
TWIST1: Drosophila homolog of 1
