# Supplementary material for: Enhanced expression of the stemness-related factors OCT4, SOX15 and TWIST1 in ectopic endometrium of endometriosis patients
Source: Reprod Biol Endocrinol. 2016 Nov 24;14:81. doi: 10.1186/s12958-016-0215-4 (PMC5122168; doi:10.1186/s12958-016-0215-4)

| PatientID | Tissue | CtACTB | CtGAPDH | CtDCAMLK1 |
| --- | --- | --- | --- | --- |
| 1 | eutopic | 20,2852459 | 20,3742599 | 31,0301075 |
| 1 | ectopic | 26,0974903 | 25,8552399 | 33,9917336 |
| 2 | eutopic | 21,9242935 | 21,3073387 | 31,9321804 |
| 2 | ectopic | 30,3871346 | 29,276762 | 39,0380363 |
| 3 | eutopic | 21,3664894 | 21,5111389 | 32,3330879 |
| 3 | ectopic | 26,5619106 | 26,7749844 | 34,8380241 |
| 4 | eutopic | 20,9857597 | 21,65172 | 31,2163563 |
| 4 | ectopic | 27,3123932 | 27,0940838 | 35,1383705 |
| 5 | eutopic | 22,4085178 | 20,634716 | 32,0083389 |
| 5 | ectopic | 28,0977554 | 26,9769974 | 35,3240814 |
| 6 | eutopic | 22,4929428 | 22,3020744 | 31,4964962 |
| 6 | ectopic | 27,8855286 | 26,61549 | 36,3181381 |
| 7 | eutopic | 22,8078461 | 24,0179634 | 31,3141136 |
| 7 | ectopic | 28,081665 | 27,1474724 | 34,3259048 |
| 8 | eutopic | 23,1817818 | 22,9186516 | 31,0197201 |
| 8 | ectopic | 24,3453655 | 24,6391525 | 34,6442757 |
| 9 | eutopic | 20,6771946 | 19,8770409 | 33,3101425 |
| 9 | ectopic | 27,0931816 | 26,2500954 | 34,9420013 |
| 10 | eutopic | 22,7088814 | 21,610014 | 32,8538857 |
| 10 | ectopic | 29,8562622 | 27,538105 | 34,8280182 |
| 11 | eutopic | 22,0171356 | 20,1928139 | 31,3921452 |
| 11 | ectopic | 26,5201759 | 27,1581688 | 34,7766037 |
| 12 | eutopic | 20,9869919 | 21,343483 | 31,4687920 |
| 12 | ectopic | 27,3736725 | 25,6270943 | 34,0113640 |
| 13 | eutopic | 24,0854015 | 22,8157501 | 32,2581749 |
| 13 | ectopic | 26,0634575 | 26,6000023 | 36,3188705 |
| 14 | ectopic | 27,4533367 | 25,6207085 | 33,5000000 |
| 14 | eutopic | 25,4851189 | 27,0913544 | 34,2756233 |
| 15 | eutopic | 20,5012836 | 21,0378952 | 33,0127716 |
| 15 | ectopic | 28,2990112 | 22,8075447 | 38,1845169 |
| 16 | eutopic | 28,8250046 | 28,0778198 | 35,3681870 |
| 16 | ectopic | 26,5136528 | 26,138586 | 36,0452652 |
| 17 | eutopic | 20,6834278 | 21,4938602 | 30,9509068 |
| 17 | ectopic | 23,4511166 | 23,7726707 | 33,0661888 |
| 18 | eutopic | 21,6647263 | 21,1889763 | 33,2259712 |
| 18 | ectopic | 28,5558624 | 25,6103897 | 36,4587593 |
| 19 | eutopic | 22,3191338 | 23,0285759 | 31,6455555 |
| 19 | ectopic | 26,3594437 | 24,759613 | 32,0595284 |
| 20 | ectopic | 24,0504837 | 24,2955246 | 30,8999462 |
| 20 | eutopic | 20,7622757 | 20,9588909 | 32,9855957 |
| 21 | eutopic | 21,6810341 | 21,3243599 | 32,0594101 |
| 21 | ectopic | 24,716444 | 23,7164612 | 32,2322769 |
| 22 | eutopic | 20,8473701 | 19,7807121 | 31,9561596 |
| 22 | ectopic | 28,5659485 | 25,7747574 | 32,8874207 |
| 23 | eutopic | 21,0951996 | 22,954071 | 32,1988907 |
| 23 | ectopic | 28,3872433 | 27,5468483 | 33,9530563 |
| 24 | ectopic | 23,8587914 | 23,3054657 | 31,3905773 |
| 24 | eutopic | 21,270546 |  | 31,7320538 |
| 25 | ectopic | 24,62043 | 23,5334206 | 31,8455315 |
| 25 | eutopic | 21,2526741 | 18,6247578 | 32,3116074 |
| 26 | ectopic | 21,4260826 | 20,2234039 | 29,5738525 |
| 26 | eutopic | 25,7867661 | 23,9633732 | 33,7414436 |
| 27 | ectopic | 22,3714638 | 22,7336025 | 30,9731846 |
| 27 | eutopic | 24,9068012 | 23,5387974 | 35,1205521 |
| 28 | ectopic | 23,2415791 | 23,5801888 | 30,1348495 |
| 28 | eutopic | 23,3426933 | 21,3916264 | 34,1933823 |
| 29 | ectopic | 25,6919193 | 24,728014 | 32,9977417 |
| 29 | eutopic | 25,8131142 | 21,8655472 | 36,3040886 |
| 30 | ectopic | 23,5288658 | 23,1015396 | 31,8675785 |
| 30 | eutopic | 23,854784 | 23,5491047 | 34,7780190 |
| 31 | ectopic | 23,3462048 | 23,3614311 | 30,8219891 |
| 31 | eutopic | 25,8297615 | 23,141819 | 34,4833717 |


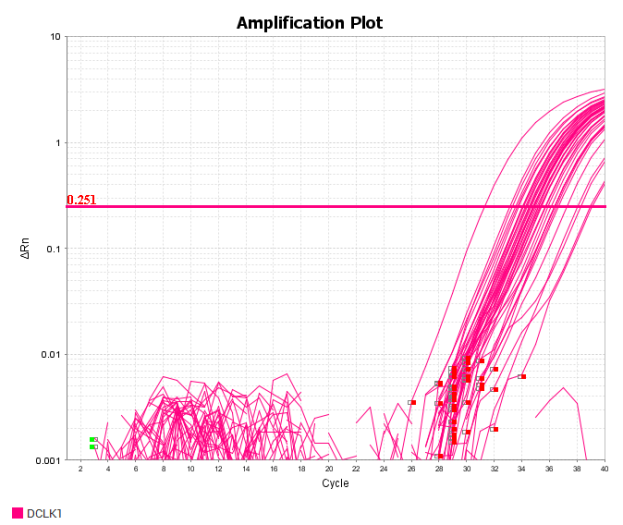

Supplement: Additional file 1: — Quantitative Real Time PCR was used to analyze the mRNA expression levels of DCAMLK1. Representative qRT-PCR results (Ct values) for DCAMLK1 and control genes (ACTB and GAPDH) are shown. (DOCX 216 kb) [file 12958_2016_215_MOESM1_ESM.docx]
